# The enhancing antibiofilm activity of curcumin on *Streptococcus mutans* strains from severe early childhood caries

**DOI:** 10.1186/s12866-020-01975-5

**Published:** 2020-09-16

**Authors:** Bingchun Li, Ting Pan, Huancai Lin, Yan Zhou

**Affiliations:** 1 Department of Preventive Dentistry, Hospital of Stomatology, Guanghua School of Stomatology, Sun Yat-sen University, 56 Ling Yuan Road West, Guangzhou, 510055 China; 2 Guangdong Provincial Key Laboratory of Stomatology, Sun Yat-Sen University, Guangzhou, China

**Keywords:** Curcumin, *Streptococcus mutans*, Clinical isolates, Biofilm

## Abstract

**Background:**

*Streptococcus mutans* (*S. mutans*) is one of the main cariogenic bacteria for caries. It was found that the clinical strains of *S. mutans* isolated from caries active population have stronger cariogenic ability than the isolates from caries-free (CF) people. Previous studies have found that curcumin can inhibit biofilm formation of *S. mutans* UA159. The objective of this study is to explore the antibiofilm effect of curcumin on the clinical isolates of *S. mutans* from severe early childhood caries(SECC).

**Results:**

The isolates from SECC group had more biomass than CF group (t = 4.296, *P* < 0.001). The acidogenicity and aciduricity of the strains from two groups showed no significant difference. After treatment with curcumin, the viability of biofilm was reduced to 61.865% ± 7.108% in SECC and to 84.059% ± 10.227% in CF group at 24 h (*P* < 0.05). The net reduction of live bacteria and total bacteria in the SECC group was significantly higher than that of the CF group (live bacteria t = 3.305, *P* = 0.016; total bacteria t = 2.378, *P* = 0.045) at 5 min. For 24 h, the net reduction of live bacteria and total bacteria in the SECC group was significantly higher than that of the CF group (live bacteria t = 3.305, *P* = 0.016; total bacteria t = 2.378, *P* = 0.045). The reduction of biofilm thickness reduced significantly in 5 min (t = 4.110, *P* = 0.015) and in 24 h (t = 3.453, *P* = 0.014). Long-term (24 h) curcumin treatment inhibited the amount of EPS in SECC group from (25.980 ± 1.156) μm^3^/μm^2^ to (20.136 ± 1.042) μm^3^/μm^2^, the difference was statistically significant (t = 7.510, *P* < 0.001). The gene of *gtfC*, *gtfD*, *ftf*, *gbpB*, *fruA* and *srtA* in the CF group and the *gtfB*, *gtfC*, *gtfD*, *ftf*, *gbpB*, *srtA* in SECC group were respectively reduced after 5 min curcumin treatment. After 24 h treatment, the *gtfB*, *gtfC*, *gtfD*, *ftf*, *gbpB*, *fruA* and *srtA* in both two groups were downregulation, all the differences were statistically significant.

**Conclusions:**

Curcumin has antibiofilm activity on clinical strains of *S. mutans*, especially for those isolated from SECC.

## Background

Dental caries, especially severe early childhood caries (SECC), severely impacts the oral health of children. SECC process rapidly, lead to destruction of the primary dentition and increase the risk of new caries lesions in the permanent dentition [1]. The American Academy of Pediatric Dentistry (AAPD) defines SECC in children aged 3–5 years as: one or more cavitated, missing (due to caries) or filled smooth surface in primary maxillary anterior teeth or decayed, missing or filled surfaces greater than or equal to four (age of 3), five (age of 4) or six (age of 5) [2]. SECC is a serious public health problem and linked to a greater financial burden in China [3].

The etiology of SECC is complex and diverse. Cariogenic bacteria plays a role in the process of dental caries by forming biofilms [4, 5]. The biofilms are highly dynamic and structured communities of microorganism that are firmly attached to teeth in a three-dimensional (3D) extracellular matrix of polymeric substances such as exopolysaccharides (EPS), proteins and nucleic acids [6, 7]. The microbial composition and structural organization of cariogenic biofilms are complex and dynamical changeable [8]. *Streptococcus mutans* (*S. mutans*) is one of the main cariogenic bacteria for caries based on its acid-producing, acid-resistant and adhesive properties [8–11]. *S. mutans* can rapidly modulate the formation of cariogenic biofilms when dietary sucrose and starch are present. Then many other organisms adhere to the biofilm and take effect [4, 12, 13]. The biofilm modulated by *S. mutans* plays a crucial role in the formation of subsequently mature and complex cariogenic biofilm. Thus, enhanced understanding of how *S. mutans* biofilms can be disrupted and removed from the surface of attachment could lead to improved strategies to eradicate them.

It was found that the clinical strains of *S. mutans* isolated from caries active population have stronger cariogenic ability than the isolates from caries-free (CF) people [14]. *S. mutans* clinical strains from different individuals have variable virulence [15]. Phenotypic traits of *S. mutans* strains would be associated with their ability to colonize tooth surface or express factors that could induce the formation of caries lesions [16]. The biological characteristic of *S. mutans* strains varied from SECC children to CF children. The strains with different biological traits would have different reaction to antibiotics.

Curcumin is a natural plant present in the *Curcuma longa* (turmeric) and has extensive clinical application [17]. It was used as an inhibitor of quorum sensing in bacteria [18].Previous studies have found that curcumin could decrease the biofilm activity of *S. mutans* UA159. It could eliminate the mature biofilm of *S. mutans* UA159 (24 h) by reducing the formation of EPS and change the ratio of dead and living bacteria [19]. Furthermore, we found that curcumin is more sensitive to *S. mutans* in dual-species biofilm [20]. Curcumin is a potential natural product in the antibacteria of *S. mutans*. It was reported that clinical isolates of *S. mutans* have different cariogenic properties [15]. Strains derived from SECC exhibited cariogenic traits and difficult to remove [21]. It is not clear whether curcumin has effect on the *S. mutans* isolated from SECC.

Therefore, the aim of present study is to explore the potential effect of curcumin on the biofilm of *S. mutans* strains isolated from SECC and provide evidence for the clinical application of natural compound.

## Results

### Different biological properties of the clinical strains of *S. mutans*

**Fig. 1:**
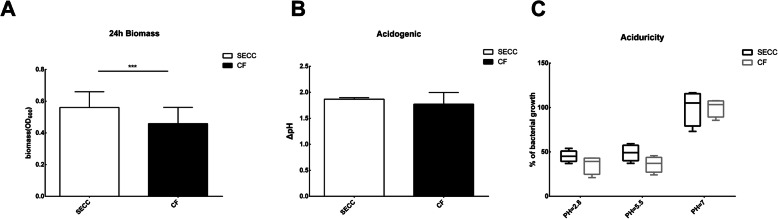
Biological properties of the clinical isolates from SECC group and CF group. Clinical strains of *S. mutans* were subjected to anaerobic culture at 37 °C to form a 24 h biofilm. The biomass of the biofilm was evaluated by crystal violet (CV) staining method. The result was showed in **a** The acidogenicity assay was used to evaluate the ability of acid-producing of the clinical strains. The reduction of the pH values of the medium was used to represent the ability of acid production of the strains. The result was showed in **b** The aciduricity assay was used to explore the ability of aciduricity of the *S. mutans* clinical strains through the percentage of bacterial growth, the result was showed in **c** The assay of the two groups were repeated three times, and the data represented the mean ± standard deviation of three independent experiments. * indicates that the data is statistically different (* *P* < 0.05, ** *P* < 0.01, ***P < 0.001)

As shown in Fig. 1a, the biomass of the clinical isolates was different between two groups. The amount of biomass in the SECC group was greater than that in the CF group, and the difference was statistically significant (t = 4.296, *P* < 0.001). As shown in Fig. 1b, the reduction of pH value was 1.868 ± 0.028 in SECC group and 1.772 ± 0.225 in CF group, there was no significant difference in the acid production ability between two groups(t = 1.272, *P* = 0.238). The aciduricity assay in Fig. 1c showed that the aciduricity of *S. mutans* in SECC group is slightly stronger than those of in CF group, but there was no significant difference between groups.

### The effect of curcumin on biofilm activity of *S. mutans* clinical strains

**Fig. 2:**
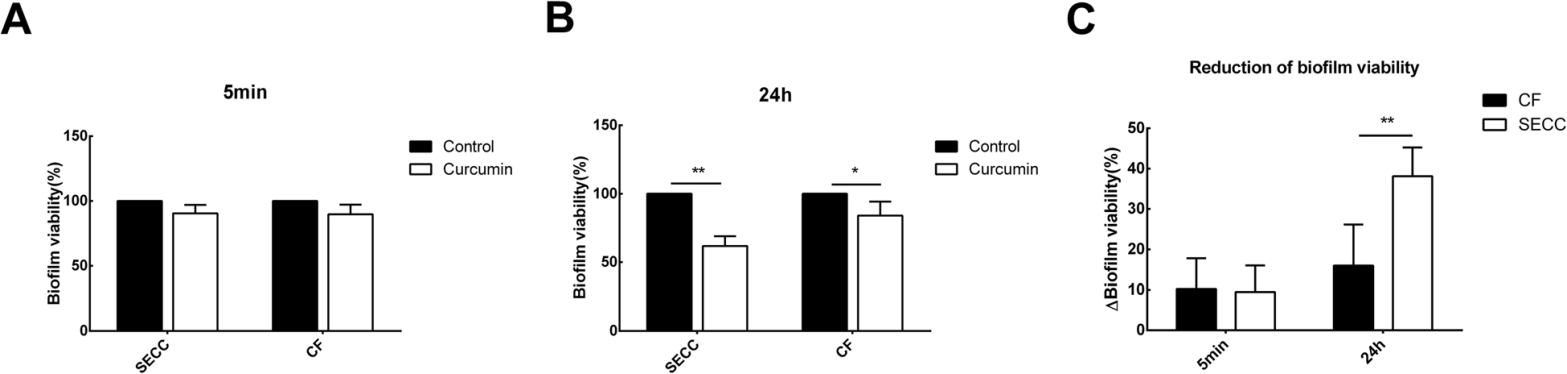
Effects of curcumin on biofilm activity of *S. mutans* in different groups at 5 min/24 h. Bacteria were inoculated in 96-well microtiter plates containing BHIS medium to form a 24-h biofilm. Supernatant was discard and it was washed with PBS for three times. The experimental group was added with 500 μM curcumin in BHIS, and the negative control group was only added with BHIS. After 5 min (**a**) and 24 h (**b**), the activity of the biofilm of the *S. mutans* was evaluated by MTT assay. Meanwhile compared the difference of reduction in biofilm viability (%) between the SECC group and CF group after 5 min and 24 h drug treatment (**c**). * indicates that the data is statistically different (* P < 0.05, ** P < 0.01, ***P < 0.001)

As shown in Fig. 2a, there was no significant effect of curcumin on the biofilm viability of the two groups after 5 min’ treatment. After long-term (24 h) action of curcumin in Fig. 2b, the biofilm activity of the SECC group was reduced to 61.865 ± 7.108%, the difference was statistically significant (t = 10.731, *P* = 0.002). The biofilm viability of the CF group decreased to 84.059 ± 10.227%, the difference was statistically significant (t = 3.485, *P* = 0.025).

Figure 2c showed the net reduction of biofilm viability after 5 min and 24 h curcumin treatment. There was no significant difference between 5 min treatment. However, the biofilm viability (%) in the SECC group decreased by 38.135 ± 1.708%, and that in the CF group decreased by 15.941 ± 1.023% at 24 h. The difference between the two groups was statistically significant at 24 h. (t = 3.832, *P* = 0.007).

### Effect of curcumin on the ration of live/dead bacteria in clinical strains of *S. mutans*

**Fig. 3:**
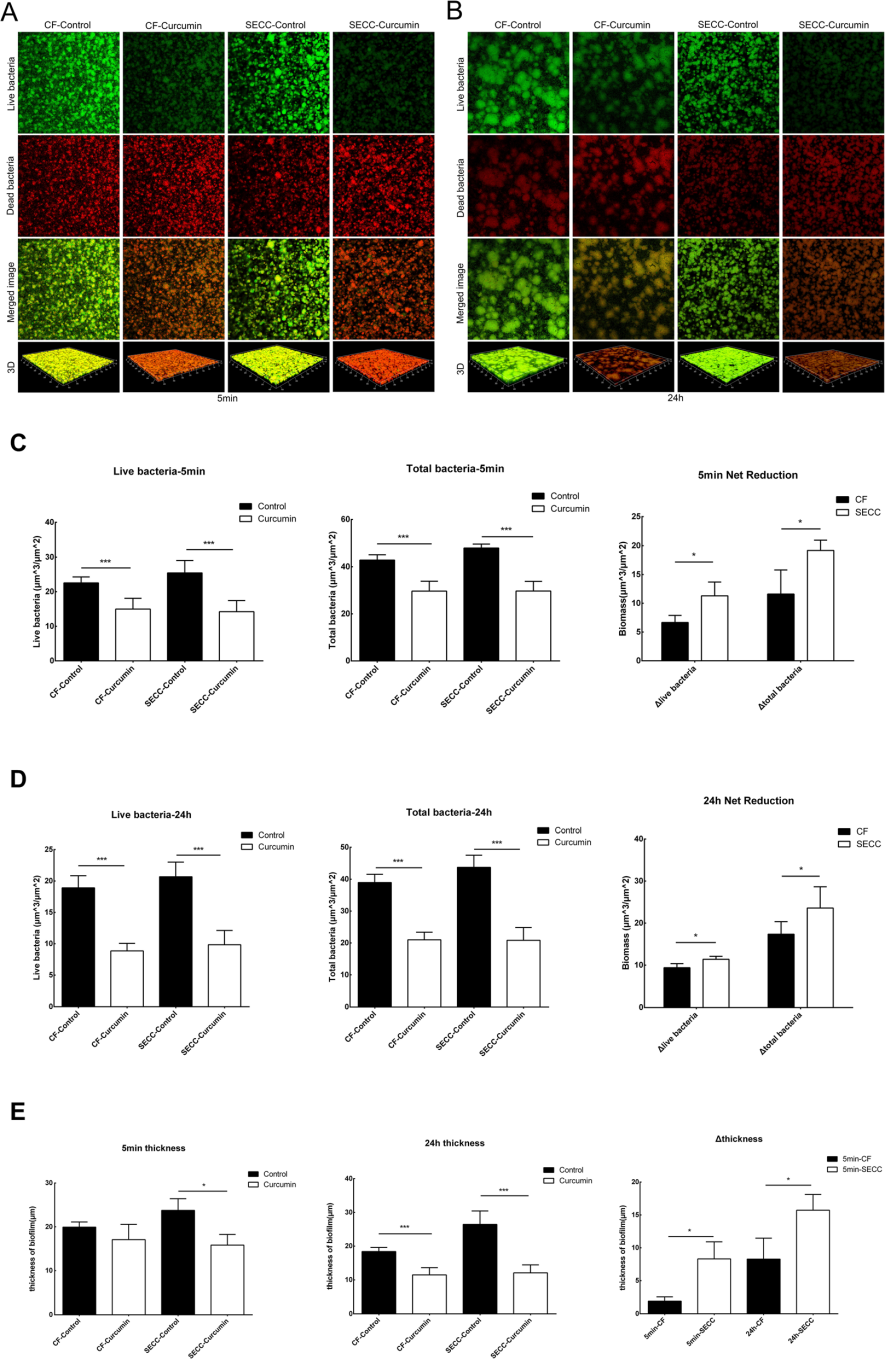
Effect of curcumin on live/dead bacteria and thickness of the biofilm of *S. mutans*. After mature biofilm formed by clinical strains, the biofilm was treated with 500 μM curcumin for 5 min (**a**) and 24 h (**b**). The green color of image was for live bacteria, red color was for dead bacteria, merged image was for dead and live bacteria, and three-dimensional reconstruction image was also showed. **c** and **d** showed the live bacteria and total bacteria counts after the treatment of curcumin. **e** showed the change of the biofilm thickness after the curcumin- treated in the SECC and the CF group. The data are expressed as mean ± standard deviation. * indicates that the data for the different groups are statistically different (* P < 0.05, ** P < 0.01, ***P < 0.001)

The images of CLSM were showed in Fig. 3. Green fluorescence represented live bacteria, red fluorescence represented dead bacteria, and yellow fluorescence was an overlap of live bacteria and dead bacteria. The image of one of the nine strains from each group was showed in Fig. 3a&b. The green fluorescence density of the curcumin-treated group was lighter than that of the control group in both SECC group and CF group. The red fluorescence density of the curcumin-treated group was much darker in curcumin-treated group than that of the control group (Fig. 3a &b).

The results of all the strains were combined and showed in Fig. 3c&d. At 5 min, the amount of live bacteria in the clinical strains of *S. mutans* decreased. The change was from (22.560 ± 1.736) μm^3^/μm^2^ to (14.999 ± 3.116) μm^3^/μm^2^ (t = 6.824, *P* < 0.001) in CF group while was from (25.460 ± 3.579) μm^3^/μm^2^ to (14.228 ± 3.237) μm^3^/μm^2^ (t = 7.726, P < 0.001) in SECC group. The total bacteria also showed the same trend. The CF group decreased from (42.841 ± 2.284) μm^3^/μm^2^ to (29.671 ± 4.197) μm^3^/μm^2^ (t = 7.509, *P* < 0.001) and the SECC group decreased from (48.007 ± 1.676) μm^3^/μm^2^ to (29.716 ± 4.101) μm^3^/μm^2^ (t = 10.304, *P* < 0.001). The net reduction of live bacteria and total bacteria in the SECC group was significantly higher than that of the CF group (live bacteria t = 3.017, *P* = 0.030; total bacteria t = 2.881, *P* = 0.045) (Fig. 3c).

After treated with curcumin for 24 h, the amount of live bacteria in the biofilm formed by the clinical strains of the CF group decreased from (18.906 ± 1.934) μm^3^/μm^2^ to (8.860 ± 1.192) μm^3^/μm^2^ (t = 17.129, *P* < 0.001). The amount of live bacteria in the biofilm formed by SECC group decreased from (20.684 ± 2.320) μm^3^/μm^2^ to (9.840 ± 2.274) μm^3^/μm^2^ (t = 12.927, P < 0.001). The amount of total bacteria also showed the descending trend. In CF group, it was decreased from (38.943 ± 2.615) μm^3^/μm^2^ to (21.005 ± 2.381) μm^3^/μm^2^ (t = 16.038, P < 0.001). In SECC group, it was decreased from (43.716 ± 3.812) μm^3^/μm^2^ to (20.839 ± 4.016) μm^3^/μm^2^ (t = 12.008, P < 0.001). The net reduction of live bacteria and total bacteria in the SECC group was significantly higher than that of the CF group (live bacteria t = 3.305, *P* = 0.016; total bacteria t = 2.378, *P* = 0.045) (Fig. 3d).

### The effect of curcumin on the thickness of biofilm of *S. mutans* clinical strains

As shown in Fig. 3e, the short-term (5 min) effect of curcumin inhibited the biofilm thickness of the clinical strains from SECC group. The biofilm thickness formed by the SECC group decreased from (23.767 ± 2.656) μm to (15.844 ± 2.424) μm (t = 4.806, *P* = 0.001). There was no difference in the strains from CF group (t = 1.903, *P* = 0.084). But, the biofilm thickness formed by the CF group was reduced from (18.400 ± 1.229) μm to (11.500 ± 2.129) μm (t = 7.937, *P* < 0.001) after long-term (24 h) effect of curcumin. SECC group decreased from (26.450 ± 3.984) to (12.075 ± 2.381) μm (t = 5.690, *P* < 0.001). The net reduction of biofilm thickness reduced significantly in short-term effect (t = 4.110, *P* = 0.015) and in long-term effect (t = 3.453, *P* = 0.014) (Fig. 3e).

### Effect of curcumin on the formation of EPS of *S. mutans* biofilms formed by clinical strains

**Fig. 4:**
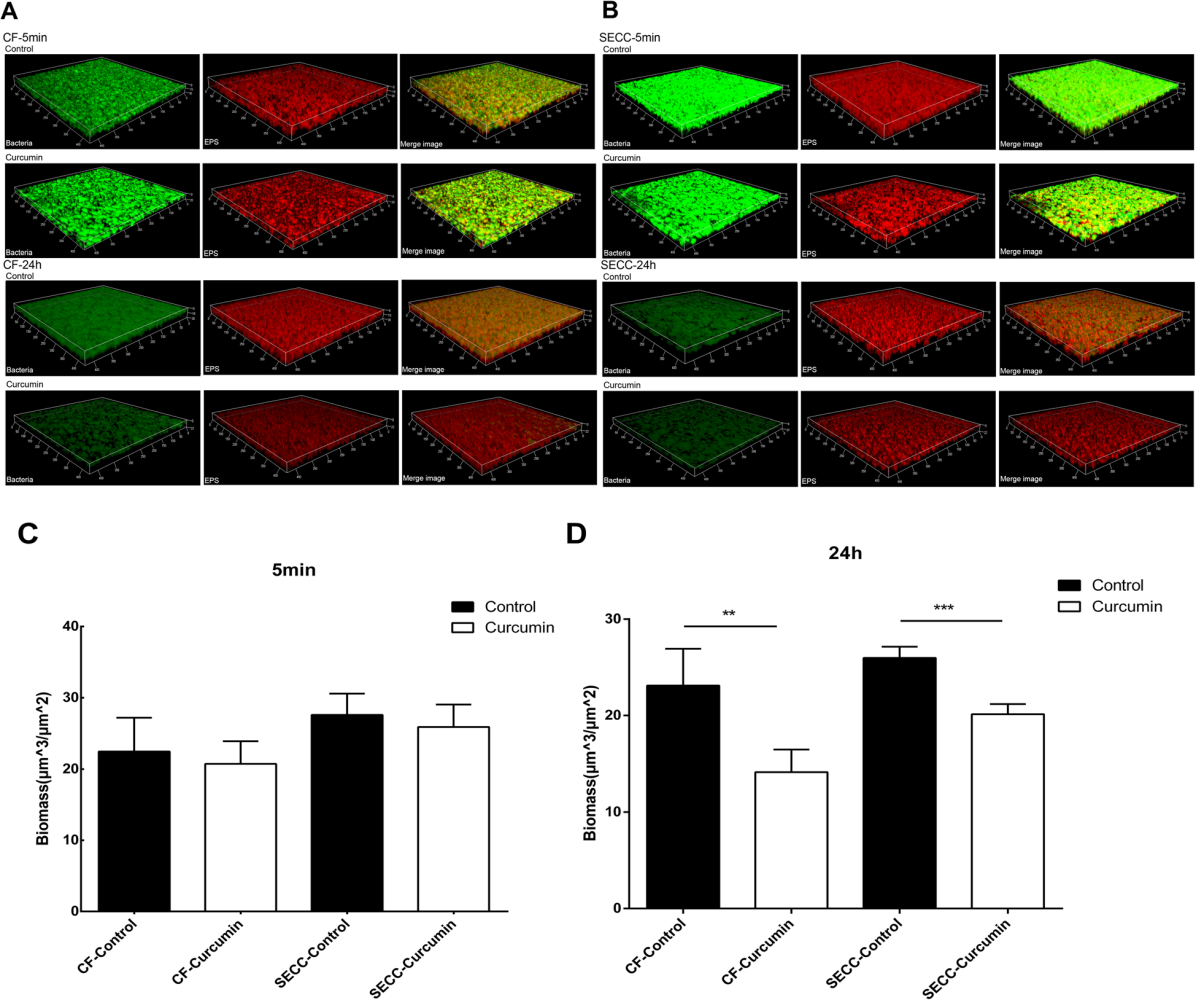
Effect of curcumin on EPS of the biofilms of *S. mutans* clinical strains. **a** and **b** show three-dimensional image reconstruction of the biofilm formed by SECC and CF group after the treatment of drug for 5 min (**a**) and 24 h (**b**), respectively. The image was scanned at 20x magnification. **c** is represented the volume of EPS after treated by curcumin for the 5-min in SECC group and the CF group. Five random fields were selected for each sample, and data was obtained from three replicates in each group. **d** shows the change in EPS in the biofilm of S. mutant clinical strain after 24 h of curcumin treatment. Data are expressed as mean ± standard deviation. * indicates statistically significant differences between the data (* P < 0.05, ** P < 0.01, ***P < 0.001)

In Fig. 4a and b, the green color represented the total bacteria, the red color represented EPS, and the yellow color was an overlap of EPS and bacteria. It can be seen that after treating with curcumin for 5 min, no inhibitory effect on the biofilm EPS can be seen in both groups (Fig. 4c). However, long-term (24 h) curcumin treatment inhibited the amount of EPS in SECC group from (25.980 ± 1.156) μm^3^/μm^2^ to (20.136 ± 1.042) μm^3^/μm^2^, the difference was statistically significant (t = 7.510, P < 0.001). There was also significant inhibition on the biomass of EPS in the CF group (t = 4.082, *P* = 0.005) (Fig. 4d).

### Effect of curcumin on biofilm formation related genes of clinical strains of *S. mutans*

**Fig. 5:**
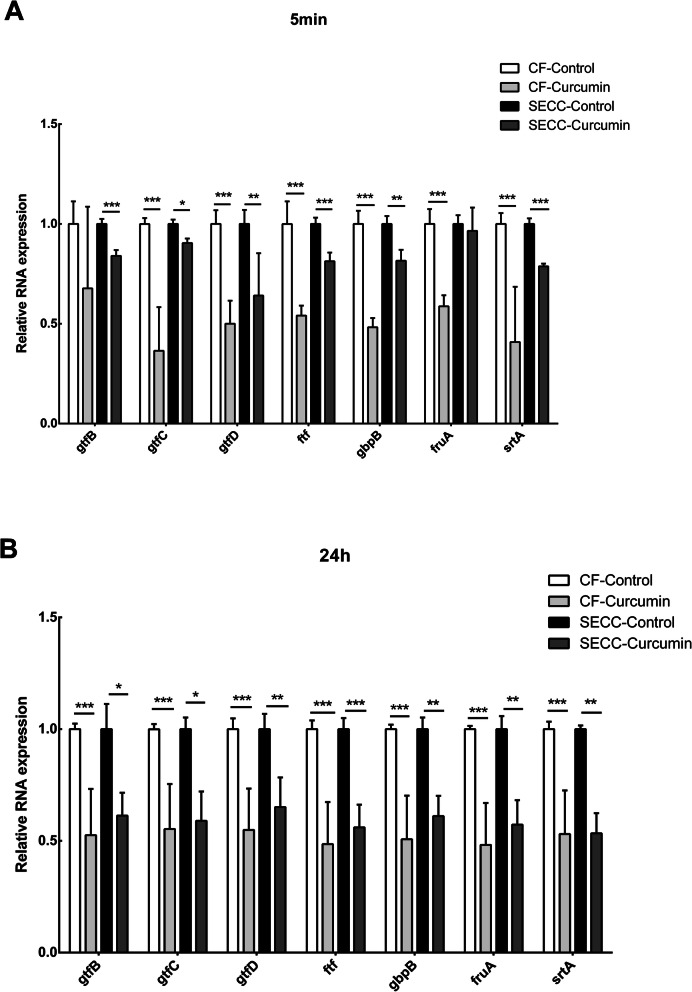
Effect of curcumin on the gene expression of *S. mutans* virulent-related factors. Gene expression levels were normalized using 16sRNA gene transcript expression levels. Data are expressed as mean ± standard deviation. * indicates statistically significant differences between the data (* P < 0.05, ** P < 0.01, *** P < 0.001)

As showed in Fig. 5, most gene expression of strains was downregulated compared to control groups. After treated by curcumin for 5 min, *gtfC*, *gtfD*, *ftf*, *gbpB*, *fruA* and *srtA* in CF group showed a reduction, which down-regulated to 0.365-, 0.501-, 0.541-, 0.482-, 0.587-, 0.408-fold. In SECC group, the *gtfB*, *gtfC*, *gtfD*, *ftf*, *gbpB*, *srtA* were respectively reduced by 0.840, 0.905, 0.641, 0.813, 0.816, 0.787 times. All the decline in both group were statistically significant (Fig. 5a).

Curcumin treatment for 24 h significantly inhibited the expression of genes related to the clinical strains of the SECC group and the CF group. Among them, *gtfB*, *gtfC*, *gtfD*, *ftf*, *gbpB*, *fruA* and *srtA* in the CF group showed a downward trend, which decreased by 0.526, 0.553, 0.549, 0.486, 0.507, 0.482, 0.530 times, all the differences were statistically significant. In the SECC group, the *gtfB*, *gtfC*, *gtfD*, *ftf*, *gbpB*, *fruA*, *srtA* down-regulated 0.530, 0.522, 0.671, 0.648, 0.674, 0.664, 0.570 times, the differences were statistically significant (Fig. 5b).

We also conducted the gene expression of quorum-sensing system of *S. mutans* biofilm (Supplemental Fig. 1). The expression of *comC* after 5-min curcumin treatment and the expression of *luxS*, *comD*, *comE*, *vicR* after 24-h curcumin treatment are significant decreased, they down-regulated 0.392, 0.380, 0.332, 0.335, 0.334 (Supplemental Fig. 1).

## Discussion

*S. mutans* is the main cariogenic bacteria of dental caries. The dental biofilm formed on the tooth surface is one of its cariogenic trait. Therefore, effective removal of the *S. mutans* biofilm can prevent and control the development caries [22]. It is well known that clinical isolates of *S. mutans* isolated from different person has variable biological characteristics. The different strains showed variable ability in cariogenicity, such as dextran-forming abilities [23], glucosyltransferases (gtfs) enzymatic activity [14]. The strain of *S. mutans* isolated from caries-active person is more capable of cariogenicity and produces more EPS [24].

In the present study, nine clinical strains from the SECC group and the CF group were respectively selected and evaluated. Though the acidogenicity and aciduricity showed no significant difference between the two groups, more biomass was formed by strains from SECC group than those from CF group. Different biomass formation showed the cariogenic ability between the two groups of clinical strains were mainly biofilms-mediated. *S. mutans* strains from SECC group had more cariogenic ability probably based on the formation of biofilm. This is consist with previous reports [23] [24].

This study found that with the treatment of curcumin for 24 h, the biofilm viability was inhibited both in the SECC and CF group. The results showed that curcumin has an inhibitory effect on the biofilm viability of clinical strains of *S. mutans*. It indicated that the long-term effect of curcumin is stronger than the short-term effect.

Then, we proceeded to explore the effect of curcumin on the live bacteria, total bacteria and thickness of biofilm by CLSM. The results revealed that curcumin was not only decreased the amount of live and total bacteria of biofilm, but also reduced the thickness of biofilm. One of the interesting finding was that more reduction of live bacteria and total bacteria was discovered on strains from SECC. The inhibitory effect on SECC group was stronger than that in CF group, indicating that curcumin may be specific on the inhibition of high biofilm-forming strains. The clinical strains of *S. mutans* from SECC formed more biomass compared to caries-free strains. Curcumin inhibited biofilm formation of *S. mutans* through disrupt of exoploysaccharides [25]. More exoploysaccharides were disrupted by curcumin on SECC group and thus inhibited biofilm formation of SECC group.

Extracellular matrices include extracellular polysaccharide (EPS), lipoteichoic acid, and nucleic acids [13]. Extracellular polysaccharide (EPS) are the main constituents of the matrix in cariogenic biofilms and are recognized as essential virulence factors associated with dental caries. The lipoteichoic acid and nucleic acids can contribute with matrix development by modulating the assembly, structural organization and functional properties of the matrix during cariogenic biofilm formation [13]. The EPS account for about 40% of the dry weight of mature biofilms, and the decrease of biofilm content may be related to the decrease of EPS in the whole biofilm matrix [26]. The results showed that curcumin significant reduced the amounts of EPS in biofilm at 24 h but not at 5 min. The EPS of clinical strains was not sensitive to curcumin in a short term which coincide with the effect on biofilm viability.

The decrease of biofilm thickness probably because the components of biofilm reduced. The amount of bacteria and of extracellular matrix secreted by bacteria inhibited by curcumin. The inhibition of bacteria and EPS in SECC group was more efficient, the reduction of thickness in SECC was more obvious. The reduction of biofilm thickness would cause the deformation of the three-dimensional structures of biofilm, which could take an effect on cariogenic of *S. mutans.*

In our study, the cariogenic ability between the two groups of clinical strains were mainly biofilms-mediated. The process of biofilm formation by *S. mutans* via two independent mechanisms: sucrose-dependent and sucrose-independent [27]. The sucrose-dependent mechanism of plaque formation is based on glucosyltransferases (GTFB, −C, and-D) produced by *S. mutans* in combination with glucan-binding proteins (GBPs) [28]. The sucrose-independent mechanism is not relevant in the virulence of *S. mutans* but in adhesion [28]. Therefore, we examined the effect of curcumin on these two pathogenic mechanism-related genes of *S. mutans*.

As the results showed, we found that curcumin may inhibit the biofilm activity of clinical strains of *S. mutans* through inhibited the expression of genes involved in biofilm formation. The formation of EPS of *S. mutans* mainly included glucosyltransferases and fructosyltransferase [29]. The glucosyltransferase (gtfs) is an essential enzyme for bacteria to utilize sucrose and form glucan, which plays an important role in the formation of biofilm and the development of dental caries [30]. Gtfs encoded by the *gtfB*, *gtfD*, and *gtfC* gene which synthesized water-insoluble glucan, soluble glucan, and the mixture [31–33]. Fructosyltransferases are primarily enzymes that convert sucrose into extracellular fructose homopolymers encoded by the *ftf* gene [34]. The expression of above genes expression degraded both in SECC and CF group.

In addition, bacteria aggregation is also an important condition for the formation of EPS, which is mediated by glucan binding protein (Gbp), which promotes the formation of plaque [35]. Previous research shows that the absence or mutation of the gene encoding GbpB results in a change of cell shape and a slowing down of its growth. This disables the appropriate development of biofilm, which becomes a product of non-regular cell clusters surrounded by a matrix of unusual structure [36]. The qPCR experiment found that curcumin inhibited the formation of EPS-related genes of the CF and SECC group clinical *S. mutans* biofilm, and it was inconsistent with the changes of EPS in the CF group. The expression of *S. mutans* associated virulence genes is a complex regulatory network. This research mainly detects changes in the expression of genes involved in the formation of EPS. The results of the decreased expression of these genes were consistent with the results of the biological characteristics of the SECC group, but were not confirmed in the CF group, indicating that curcumin may be different for CF and SECC group *S. mutans* clinical strains. The results of quorum-sensing system showed that Curcumin has little inhibitory effect on the genes of SECC and CF group after 5 min, while it can effectively inhibit related genes in SECC group after 24 h. However, curcumin’s inhibitory effect on the quorum-sensing system is not as obvious as that of the glucosyltransferases at short term time point. It proved that other gene regulatory systems may also be involved in mediating the effect of curcumin. The complex regulatory mechanism leads to differences in its inhibitory effects, and further research is needed. All the above experiment results indicated that after the effect of curcumin, the gene expression of glucosyltransferases and the amount of bacteria were reduced, which further decreased of EPS production and lessened of biofilm thickness. Our experiment results showed that curcumin could inhibit biofilm formation in SECC group through inhibit EPS production. Moreover, the longer curcumin acts, the more obvious its inhibitory effect. The results suggested that curcumin can effectively inhibit the cariogenicity of *S. mutans* biofilm. The stronger long-term effect of curcumin reminds us that in subsequent clinical applications, in addition to applying curcumin to short-term antibacterial products such as mouthwash, it can also be added to dental materials with slow release effects.

As previous reported showed, the eDNA and LTA also play a crucial role in the formation of *S. mutans* biofilm [13].We would continue to explore the relevant mechanism. Furthermore, how gene changes work through protein regulation. How curcumin inhibits the reduction of the number of *S. mutans* bacteria, whether there are other pathways that curcumin inhibit the cariogenic of *S. mutans* biofilm, still need to be further explored.

In summary, curcumin has antibiofilm activity on clinical strains of *S. mutans*, especially for those isolated from SECC.

## Conclusion

Curcumin has variable effect on the different clinical isolates. It has more effective long-term anti-biofilm activity in SECC clinical strains than CF clinical strains.

## Methods

### Clinical strains

The clinical isolates of *S. mutans* were derived from a previous study [37]. *S. mutans* strains were isolated from children without caries (CF group) and children with a DMFT index of ≥6 (SECC group). A subset of nine *S. mutans* clinical strains were randomly selected from the SECC and CF group separately. In total, eighteen isolates of *S. mutans* were used in the next step.

### Growth condition of clinical strains

The clinical isolates were inoculated on the brain heart infusion broth (BHI) medium and incubated in 37 °C, 85% N_2_, 5% CO_2_, 10% H_2_ anaerobic culture, observed after 24 h. After the bacterial morphology was confirmed to be pure culture, the single colony was collected and incubated into the BHI culture solution. After overnight culture, the bacterial solution was normalized to OD ≈ 0.5 (10^8^ colony forming units per milliliter (CFU/ml)), and diluted 10-fold (10^7^ CFU/ml) for the experiment.

### The biological characters of the clinical strains of *S. mutans*

#### Biomass of the clinical strains of *S. mutans*

Crystal violet staining were used to evaluate the biomass of clinical strains [38, 39]. Clinical strains from two groups were cultured in 96-well flat plates for 24 h to form biofilm. The medium is BHI with 1% sucrose (1% BHIS). Then, the contents of the microplate were removed and the wells were washed with PBS, fixed with 95% methanol, washed again and stained with 0.1% (wt/vol) crystal violet solution for 15 min at room temperature. Subsequently, the microplates were vigorously tapped on napkins to remove any excess liquid and air-dried. The remaining CV was dissolved in 100 μl absolute ethanol for 15 min at room temperature, and finally, 75 μl from each sample was transferred to a new 96-well plate, and the extract was read at 600 nm in a spectrophotometer [19, 38, 39]. The experiments were repeated for three times independently.

#### Acidogenicity of the clinical strains of *S. mutans*

The bacterial suspensions of the SECC group and the CF group were sequentially inoculated into the BHI medium (1:100). The pH value of the medium was measured before the growth of the strains by the pH meter (METTLER TOLEDO, Switzerland) and was set as pH 0. Cultured under anaerobic conditions for 48 h at 37 °C, the culture was centrifuged at 4 °C, 3000 r / min for 15 min and the supernatant medium was taken. The pH value of the supernatant was measured and was set as pH 1. The reduction of the pH values of the medium was used to represent the ability of acid production of the strains. The calculation was ΔpH = pH 0 - pH 1 [40]. Each sample was taken in triplicate. The acidogenicity of two groups was assessed.

#### Aciduricity of the clinical strains of *S. mutans*

The aciduricity was acid tolerance ability of clinical strains under acidic environment. The clinical strains of *S. mutans* was resuscitated and cultured overnight. The bacterial solution was normalized to OD ≈ 0.5 (10^8^ colony forming units per milliliter (CFU/ml)), and diluted 10-fold (10^7^ CFU/ml) for the experiment. Then, the medium was changed for fresh acidic BHI medium. The pH value of fresh BHI medium was set at pH = 2.8, 5.5 and 7.0 for acid pressure stimulation (50 mM KCl and 1 mM MgCl2, 0.1 M glycine buffer). The mixture was continuously agitated at room temperature. The medium at 0 (T0) and 60 min (T60) was collected and spread with BHI plates. The number of bacteria CFU was calculated after anaerobic incubation of the BHI plate for 48 h as detailed previously [21, 41, 42]. Bacteria viability at each time point was expressed as the percentage of bacterial growth in relation to T_0_ (100%). Analyses were performed in triplicate.

### The effect of curcumin on the clinical strains of *S. mutans*

#### Biofilm metabolism assessment by MTT assay

The 500 μM concentration of curcumin was prepared from the powder of curcumin (Sigma, USA), which stocked in dimethyl sulfoxide (DMSO) with 250 mM concentration and was diluted in BHI. The bacterial strains of the SECC group and the CF group were added to a 96-well flat plate, cultured at 37 °C, 5% CO_2_ incubator in BHIS to form 24 h biofilm. The biofilm was rinsed twice with sterile physiological saline (PBS).

The fresh medium contained 500 μM curcumin was used in the experiment. The negative control was the curcumin-free medium with bacterial solution and the blank control was the curcumin medium without bacterial solution. Both groups have their own negative control wells and blank control wells. Then, after incubated for 5 min and 24 h at 37 °C, 5% CO_2_ incubator, thiazolyl bromide at a final concentration of 0.5 mg/ml was added and incubated in the dark at 37 °C, 5% CO_2_ incubator for 3 h. The supernatant of the plates was discard and rinsed twice with PBS. 100 μl absolute DMSO was added into wells and 75 μl from each wells were transferred to a new 96-well plate. The extract was read at 570 nm in a spectrophotometer. The biofilm viability (%) = (experimental absorbance value-blank absorbance value) / (negative control absorbance value-blank absorbance value) × 100%.

#### Analysis the ratio of live and total bacteria and the thickness of biofilm by CLSM

Confocal Laser scanning microscopy (CLSM) was used to detect the ratio of dead and live bacteria and the biofilm thickness after curcumin treatment in clinical strains of *S. mutans*. Formed biofilm in confocal culture dish was incubated with curcumin for 5 min and 24 h. After washed with PBS, the dye stained with live and dead bacteria was added into plates and incubated at room temperature for 15 min. The dye was from L-7012 LIVE/DEAD® *BacLight*™ Bacterial Viability Kits (Molecular Probes, Eugene, OR, USA). Then, biofilm washed with PBS for three times, and captured using a laser scanning microscope to capture biofilm images through ImageJ software (Bitplane, Switzerland) provides a three-dimensional image of the biofilm. Biomass (mm^3^ / mm^2^) for each channel was calculated by COMSTAT, and the proportion of dead bacteria and biofilm thickness can be calculated.

#### Analysis extracellular polysaccharides of clinical strains of *S. mutans*

The sample was added to the confocal culture dish, and the concentration of 1 μM Alexa Fluor® 647-labeled dextran conjugate red fluorescent dye was added. The biofilm was formed in the 5% CO_2_ incubator for 24 h at 37 °C. After washing the biofilm with PBS, biofilm was stained with green fluorescent SYTO9 and incubated at room temperature for 15 min; using a CLSM to capture the organism Membrane images, three-dimensional images of biofilms were provided by Image 7.0 software. The biofilm biomass (mm^3^/mm^2^) of each channel was calculated by COMSTAT, and the amount of extracellular polysaccharides (EPS) was calculated.

#### Analysis gene expression by real-time PCR

The expression of *S. mutans*-associated virulence factors was evaluated by real-time quantitative PCR. Total RNA was extracted by ultrasonic crushing and using the RNeasy Mini Kit (QIAGEN, Valencia, CA, USA), determined purity and RNA concentration by NanoDrop 2000 spectrophotometer (Thermo Fisher Scientific, Pittsburgh, PA, USA), reverse transcription and real-time quantitative PCR were performed, and the change in fold expression of the relevant gene was calculated using 2^-ΔΔCT^ [19].

### Statistical analysis

Each experiment was repeated three times independently. Statistical analysis was performed using SPSS 17.0. The data were assessed to determine whether they were normally distributed. The t-test was used to test the difference between the control- and the experimental-group. The difference was statistically significant at *P* < 0.05.

## Supplementary information

**Additional file 1: Fig. 1.** Effect of curcumin on the gene expression of quorum-sensing system. * indicates statistically significant differences between the data (* P < 0.05, ** *P* < 0.01, *** *P* < 0.001).

## Data Availability

Yes, available from corresponding author on request.

## Abbreviations

*S. mutans*: *Streptococcus mutans*
CF: Caries-free
SECC: Severe early childhood caries
AAPD: The American Academy of Pediatric Dentistry
DMFT: Decayed, missing and filled teeth
CFU: Colony forming units
BHI: Brain heart infusion broth
CV: Crystal violet
MTT: 3-(4,5-dimethyl-2-thiazolyl)-2,5-diphenyl-2-H-tetrazolium bromide
DMSO: Dimethyl sulfoxide
PBS: Physiological saline
CLSM: Confocal Laser scanning microscopy
PCR: Polymerase Chain Reaction
EPS: Extracellular polysaccharide
gtfs: Glucosyltransferase
gbp: Glucan binding protein
fru: Fructosyltransferase
