# Supplementary figures and images for: The enhancing antibiofilm activity of curcumin on Streptococcus mutans strains from severe early childhood caries
Source: BMC Microbiol. 2020 Sep 16;20:286. doi: 10.1186/s12866-020-01975-5 (PMC7493841; doi:10.1186/s12866-020-01975-5)

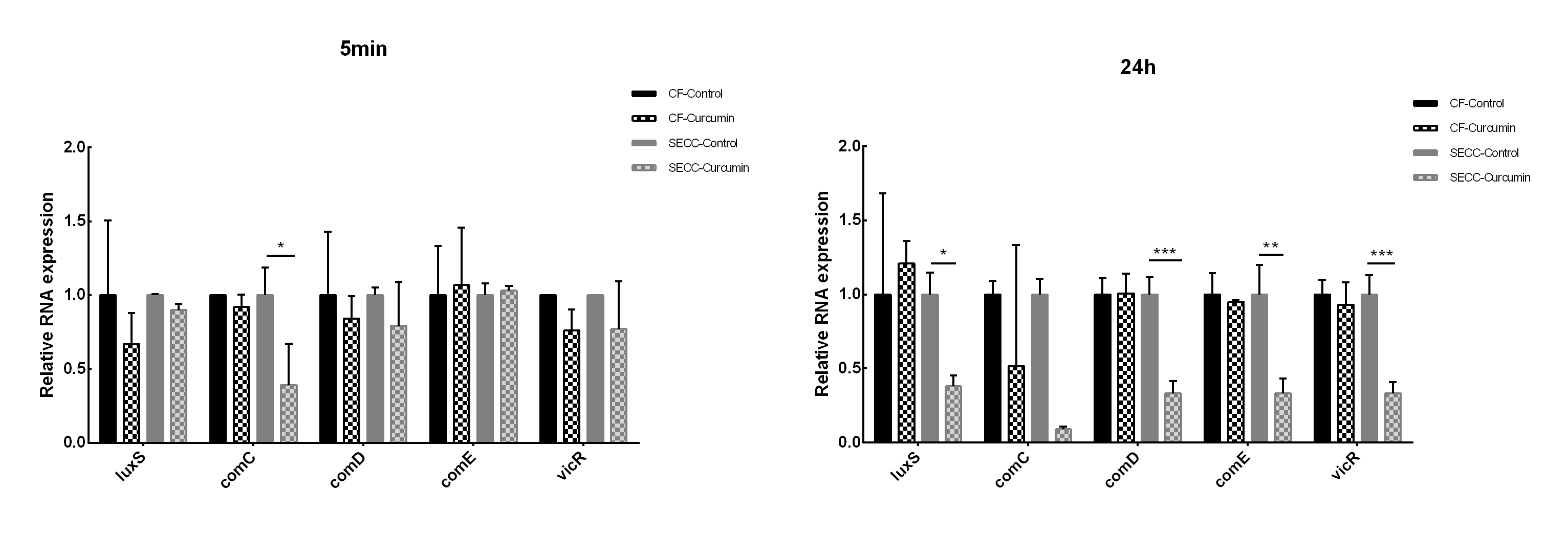

Supplement: Supplementary file 1 — Additional file 1: Fig. 1. Effect of curcumin on the gene expression of quorum-sensing system. * indicates statistically significant differences between the data (* P < 0.05, ** P < 0.01, *** P < 0.001). [file 12866_2020_1975_MOESM1_ESM.jpg]
